# Evaluation of Outcomes in Patients with Previous Stroke History following Cardiac Surgery: A Single-Center Study

**DOI:** 10.3390/jcm13144045

**Published:** 2024-07-10

**Authors:** Jerzy Pacholewicz, Paweł Walerowicz, Aleksandra Szylińska, Jakub Udzik, Paweł Wańkowicz, Ewelina Kuligowska, Ewelina Szuba, Mariusz Listewnik

**Affiliations:** 1 Department of Cardiac Surgery, Pomeranian Medical University, 70-111 Szczecin, Poland; jerzy.pacholewicz@pum.edu.pl (J.P.); pawel.walerowicz@pum.edu.pl (P.W.); jakub.udzik@pum.edu.pl (J.U.); ewelinabkulig@gmail.com (E.K.); sindbaad@poczta.onet.pl (M.L.); 2 Department of Social Medicine and Public Health, Pomeranian Medical University, 70-103 Szczecin, Poland; pawel.wankowicz@pum.edu.pl; 3 Student Science Club at the Department of Cardiac Surgery, Pomeranian Medical University, 70-111 Szczecin, Poland; ewelinaszuba01@gmail.com

**Keywords:** stroke, cardiac surgery, complications after cardiac surgery, mortality

## Abstract

**Background**: The aim of the study to analyze the risk of complications, including neurological sequelae, alongside early and late mortality among patients with antecedent stroke subjected to cardiac surgical interventions with extracorporeal support. **Material and methods**: A single-center retrospective study was conducted on 10,685 patients who underwent cardiac surgery with extracorporeal circulation at the Department of Cardiac Surgery. The first group comprised all patients eligible for cardiac surgery with extracorporeal circulation. The second group consisted of patients with a preoperative history of stroke. **Results**: In the study, a statistically significant association was observed between preoperative stroke and the occurrence of postoperative pneumonia (OR = 1.482, *p* = 0.006), respiratory failure (OR = 1.497, *p* = 0.006), renal failure (OR = 1.391, *p* = 0.019), 30-day mortality (OR = 1.528, *p* = 0.026), 90-day mortality (OR = 1.658, *p* < 0.001), and one-year mortality (OR = 1.706, *p* < 0.001). **Conclusions**: Patients with a history of preoperative stroke more frequently experienced renal failure and respiratory-system complications such as pneumonia and respiratory failure. The survival time of patients with a history of preoperative stroke was shorter compared to that of the control group during the analyzed 30-day, 90-day, and one-year observation periods.

## 1. Introduction

As the population’s longevity increases, a growing number of cardiac surgical interventions are carried out on elderly people burdened with multiple comorbidities, including a history of ischemic stroke.

Postoperative ischemic stroke is a significant complication, substantially elevating mortality rates and undermining quality of life. The risk of ischemic stroke is estimated at 1–3% following isolated coronary artery bypass grafting (CABG) and at 5–10% in more complex surgical scenarios, including aortic interventions [1]. Besides protracting hospitalization, ischemic stroke results in increased treatment expenditures, which are frequently exacerbated by the need for continuous post-discharge patient monitoring, thus underscoring its profound medical and socioeconomic significance. 

Ischemic stroke manifests predominantly in two temporal domains. Intraoperative strokes are predominantly associated with cerebral-perfusion disturbances attributable to carotid artery atherosclerosis, low-cardiac-output syndrome, and embolic events consequent to surgical manipulations involving the aortic and cardiac structures. The second period of stroke incidence pertains to the postoperative period, wherein it is primarily associated with atrial fibrillation and extant cerebrovascular pathologies [2].

Preoperative vascular interventions in asymptomatic patients used in order to mitigate the incidence of ischemic strokes remain a subject of debate. While the available guidelines offer clear directives concerning symptomatic carotid artery disease, recommendations concerning asymptomatic individuals with substantial carotid artery alterations necessitate meticulous scrutiny to determine optimal medical management strategies [3].

The established risk determinants for ischemic stroke include age, carotid artery atherosclerosis, hypertension, diabetes mellitus, atrial fibrillation, prolonged duration of extracorporeal circulation, and antecedent cerebrovascular incidents [4]. The optimal timing for non-cardiac surgical interventions following an ischemic stroke has been defined as a 9-month interval post-incident [5,6]. However, studies show discrepancies regarding the optimal interim between ischemic stroke occurrences and cardiac surgery with extracorporeal circulation. For patients with indications for surgery on an expedited/urgent basis, a 48-h period is deemed acceptable. Nonetheless, deferring non-urgent surgeries to a minimum of 4 weeks reduces the risk of neurological sequelae [7]. In general, finding the optimal timing for surgical interventions and appraising the escalated risk profile for subsequent ischemic events in individuals with antecedent stroke is a crucial issue in cardiac surgery.

This study aims to scrutinize the risk of complications, including neurological sequelae, and early and late mortality among patients with antecedent stroke subjected to cardiac surgical interventions with extracorporeal support.

## 2. Material and Methods

A single-center retrospective study was conducted on 10,685 patients who had undergone cardiac surgery with extracorporeal circulation at the Department of Cardiac Surgery of the University Clinical Hospital No. 2 of the Pomeranian Medical University in Szczecin between 2010 and 2021. Table 1 outlines the inclusion and exclusion criteria for the study. According to the study protocol, patients were excluded if they had undergone urgent surgery (620 cases) or emergency surgery (139 cases) or if they had undergone other complex cardiac surgical procedures (97 cases). All patients included in this study were self-sufficient in terms of satisfying their basic needs and required at most minor assistance in activities of daily life.

The analysis included a group of 9829 patients who had undergone cardiac surgery with extracorporeal circulation according to the study’s inclusion criteria.

### 2.1. Collecting Data

Demographic and comorbidity data were obtained through medical interviews and analysis of patient medical records. The severity of angina pectoris was assessed using the CCS (Canadian Cardiovascular Score) scale. EuroScore Logistics 2 was utilized to evaluate perioperative risk. Based on measurements of height and weight taken during the hospital stay, the BMI for each patient was calculated using the standard formula: BMI = body mass [kg]/height^2^ [m].

Postoperative-course data analyzed included intubation time, length of hospitalization, and stay in an intensive care unit, as well as in-hospital, 30-day, and long-term mortality (one-year follow-up). The occurrence of postoperative complications (cardiac, pulmonary, renal, cerebral, and infectious) was also evaluated.

### 2.2. Study Group

**Figure 1:**
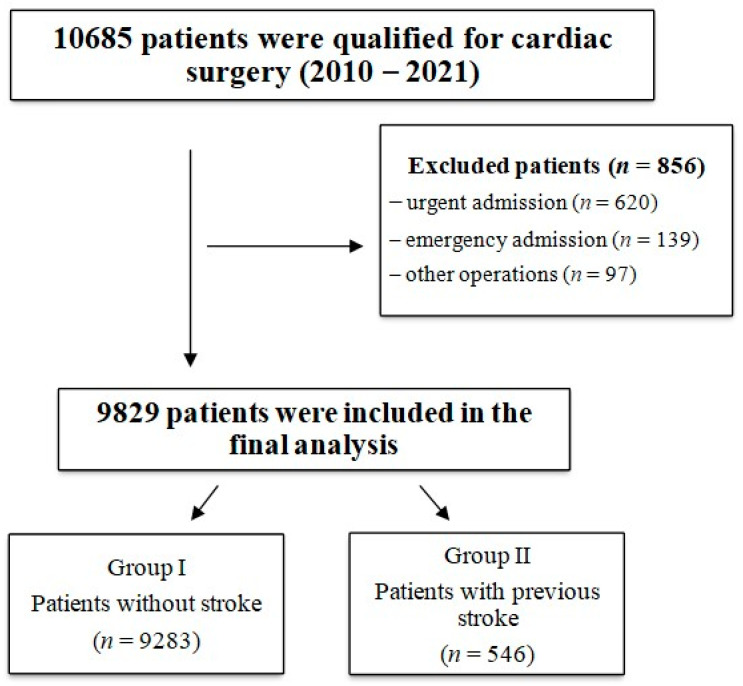
The study groups.

The study population was divided into two groups. The first group (*n* = 9283) comprised all patients eligible for cardiac surgery with extracorporeal circulation. The second group (*n* = 546) consisted of patients with a preoperative history of stroke (diagnosed according to ICD-10). The division into groups is presented in Figure 1. 

### 2.3. Ethical Issues

The study adhered to the principles outlined in the Declaration of Helsinki and Good Clinical Practice. It received an exemption from the Bioethical Committee of the Pomeranian Medical University due to its retrospective observational nature (decision no. KB.006.131.2023), Prior to surgery, each patient provided written informed consent for the procedure and anesthesia, which included consent for the collection of personal data. To ensure patient anonymity, data analysis was conducted in an anonymized form.

### 2.4. Statistical Analysis

The data were analyzed using licensed Statistica 13 software (StatSoft Inc., Tulsa, OK, USA). The normality of variable distribution was assessed using the Shapiro−Wilk test. Categorical variables were presented as numbers and percentages, and between-group comparisons were performed using the Chi-square test; Yates’s correction was applied if subgroup sizes were inadequate. Continuous variables were expressed as means, standard deviations, medians, and quartiles, with characteristics compared between patients with and without a stroke history using the Mann−Whitney U test. Single-factor and multiple-factor logistic regression analyses were conducted, with the latter adjusted for confounding variables (age, EF, ESL, comorbidities). Kaplan−Meier analysis was employed to estimate survival probability. Statistical significance was defined as *p* < 0.05.

## 3. Results

Table 2 presents demographic data, comorbidities, and intraoperative data in the patients with and without a history of preoperative stroke. Patients eligible for surgery following a stroke incident were older (*p* = 0.003), exhibited higher operative risk (*p* < 0.001), and had lower ejection fractions (*p* = 0.001). Stroke patients more frequently presented with NYHA class III and IV heart failure (*p* = 0.028), ICA stenosis (*p* < 0.001), ICA occlusion (*p* < 0.001), prior carotid artery endarterectomy (*p* < 0.001), diabetes treated with oral medications (*p* < 0.001) or insulin (*p* < 0.001), arterial hypertension (*p* = 0.032), atrial fibrillation (*p* < 0.001), and extracardiac arteriopathy (*p* < 0.001). Intra- and postoperative data revealed differences in intubation time and reintubation rates. Patients with a history of preoperative stroke experienced significantly prolonged postoperative ventilation (*p* < 0.001) and were notably more likely to require reintubation (*p* < 0.001).

Patients with a history of preoperative stroke had higher preoperative (*p* < 0.001) and postoperative (*p* < 0.001) creatinine levels. Conversely, significantly lower values were observed preoperatively for RBC (*p* = 0.049), hemoglobin (*p* < 0.001), and hematocrit (*p* < 0.001). These data are presented in Table 3.

Table 4 presents the number of complications based on the occurrence of postoperative stroke. A greater number of patients with a history of preoperative stroke after surgery exhibited delirium (21.3% vs. 15.7%; *p* < 0.001), respiratory failure (13.2% vs. 6.8%; *p* < 0.001), pneumonia (15.8% vs. 8.8%; *p* < 0.001), renal failure (14.8% vs. 8.8%; *p* < 0.001), 30-day mortality (8.1% vs. 4.2%; *p* < 0.001), 90-day mortality (11.9% vs. 5.8%; *p* < 0.001), and 1-year mortality (16.1% vs. 7.9%; *p* < 0.001).

Logistic regression analysis was performed, and the results are presented in Table 5. The table displays both single-factor and multifactorial models for risk of postoperative complications. In the multifactorial assessment adjusted by ESL, sex, and comorbidities, a statistically significant association was demonstrated between preoperative stroke and the occurrence of postoperative pneumonia (OR = 1.482, *p* = 0.006), respiratory failure (OR = 1.497, *p* = 0.006), renal failure (OR = 1.391, *p* = 0.019), 30-day mortality (OR = 1.528, *p* = 0.026), 90-day mortality (OR = 1.658, *p* < 0.001), and one-year mortality (OR = 1.706, *p* < 0.001).

Significant complications were analyzed in patients with a history of preoperative stroke undergoing CABG surgery according to the type of cardiac surgery performed (Table 6), and the results show significantly higher risks of delirium (OR = 1.425, *p* = 0.015), TIA (OR = 4.127, *p* = 0.005), EPI (OR = 3.609, *p* = 0.010), respiratory failure (OR = 2.215, *p* < 0.001), pneumonia (OR = 2.178, *p* < 0.001), and renal failure (OR = 1.839, *p* = 0.002), as well as 30-day mortality (OR = 2.290, *p* = 0.001), 90-day mortality (OR = 2.517, *p* < 0.001), and 1-year mortality (OR = 2.430, *p* < 0.001). Patients undergoing complex coronary artery bypass grafting (CABG) surgery and those with valvular defects and preoperative stroke had significantly higher risks of myocardial infarction (OR = 2.047, *p* = 0.043), respiratory failure (OR = 2.443, *p* = 0.001), pneumonia (OR = 1.675, *p* = 0.045), renal failure (OR = 1.720, *p* = 0.021), 30-day mortality (OR = 2.226, *p* = 0.006), 90-day mortality (OR = 2.106, *p* = 0.004), and 1-year mortality (OR = 2.242, *p* = 0.001). Patients with valvular defects and preoperative stroke had increased risks of postoperative stroke (OR = 2.052, *p* = 0.042) and RIND (OR = 8.172, *p* = 0.013). Patients undergoing aortic aneurysm surgery had the highest risk of extensive stroke (OR = 4.387, *p* = 0.048), renal failure (OR = 2.372, *p* = 0.040), 90-day mortality (OR = 3.179, *p* = 0.006), and 1-year mortality (OR = 2.486, *p* = 0.030).

**Figure 2:**
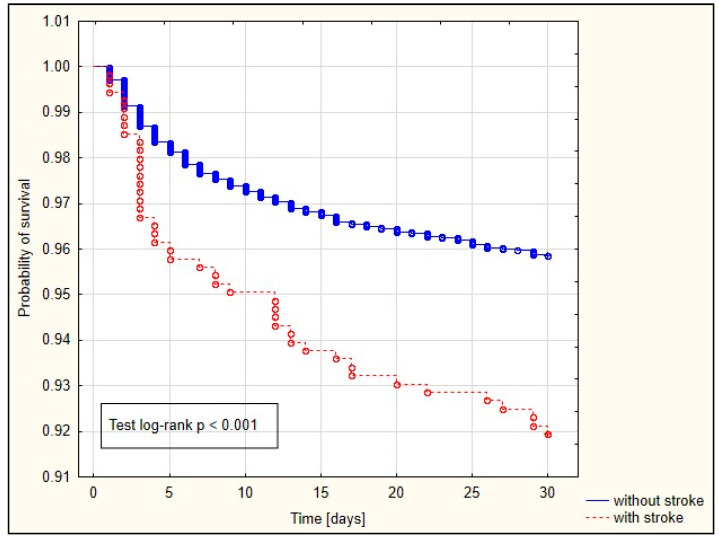
Kaplan−Meier curve illustrating the probability of survival in in patients with and without a history of preoperative stroke (30-day).

**Figure 3:**
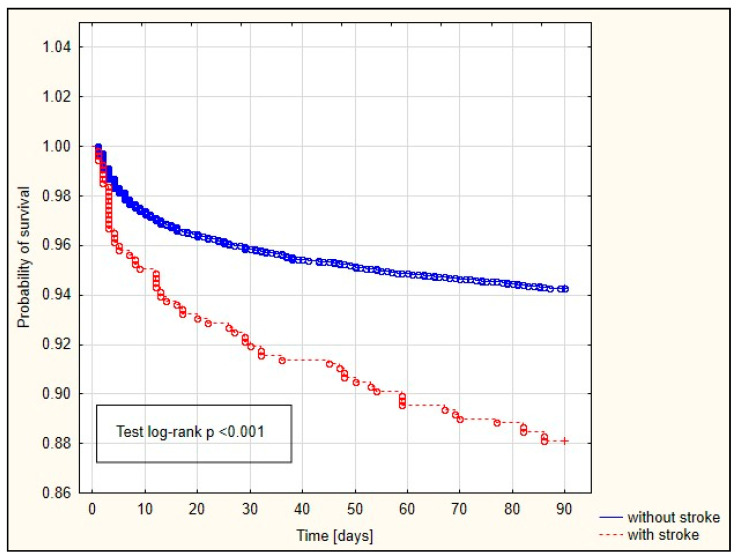
Kaplan−Meier curve depicting the probability of survival in patients with and without a history of preoperative stroke (90-day).

**Figure 4:**
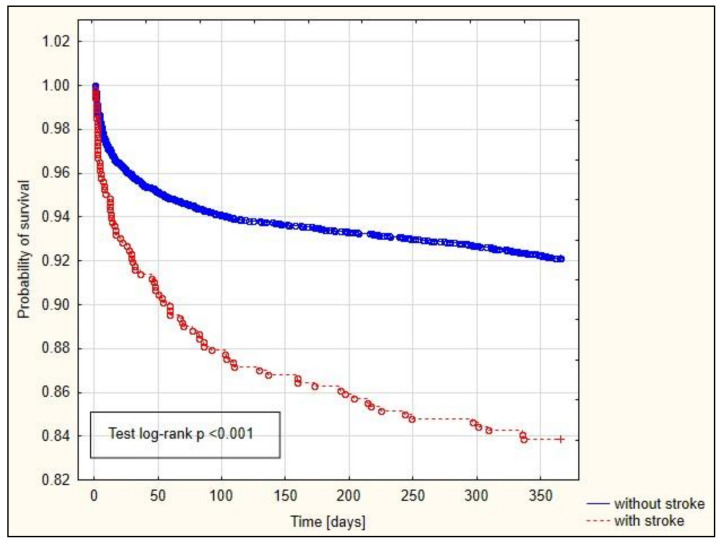
Kaplan−Meier curve illustrating the probability of survival of patients based on the presence of preoperative stroke (one-year).

The Kaplan−Meier curves are depicted in Figure 2, Figure 3 and Figure 4. Among patients with a history of preoperative stroke, significantly shorter survival times at 30 days, 90 days, and one year were observed.

## 4. Discussion

Considering the increasing prevalence of elderly patients with multiple comorbidities undergoing cardiac surgeries, it is crucial to assess perioperative risk and treatment outcomes for this demographic. In our investigation, we focused on examining how preoperative cerebral stroke influences the perioperative risk associated with on-pump cardiac surgery and subsequent treatment outcomes in these patients. A significant finding of our study was that preoperative cerebral stroke elevates the risk of postoperative complications and negatively impacts patients’ survival rates post-surgery. A cohort of 9829 patients included in this study was hospitalized in our facility over the course of eleven years (2010–2021). There were no significant changes to operating techniques or to the equipment used during the surgery in our facility during this time. 

Notably, the existing EuroScore II formula utilized to estimate cardiac-surgery risk does not incorporate cerebral stroke [8]. Messerotti Benvenuti et al., in their analysis of EuroScore and Stroke Index in cardiac-surgery patients, emphasize the necessity of assessing preoperative cognitive function, anxiety, and depression among patients scheduled for cardiac surgery. They suggest that psychological assessments should be tailored according to patients’ age and education level [9]. In our study, we observed substantial differences in EuroScore outcomes, with patients who had experienced preoperative stroke exhibiting higher scores and experiencing increased complication rates. Chang et al., assessing frailty through slow gait speed, demonstrated an over two-times-greater risk of in-hospital mortality and significantly elevated risk of complications following cardiac surgery (acute kidney injury, prolonged intubation, sternal-wound infection, prolonged ICU stay) compared to individuals with normal gait speed. They suggest that frailty assessment should be a routine part of preoperative evaluation in cardiac surgery [10].

Our study’s results indicate that prior cerebral stroke significantly influences overall operative outcomes and should be factored into patient qualification for surgery and risk−benefit assessment of such interventions. We identified an increased risk of postoperative renal failure (OR = 1.391) among patients with a history of preoperative stroke. Similarly, O’Neil et al. list previous stroke as one of the risk factors for acute kidney injury [11], a finding supported by Shurle et al. [12].

In our patient cohort with a history of preoperative stroke, individuals required prolonged mechanical ventilation and were more frequently reintubated due to respiratory failure in the early postoperative period. This led to a higher incidence of pneumonia (OR = 1.482) and respiratory failure (OR = 1.497). Our results support the findings of Knapik et al., who analyzed the impact of preoperative risk factors on risk of prolonged ventilation and found that patients with a history of preoperative stroke or TIA had a higher risk of prolonged ventilation (>48 h) [13]. Similar results were presented by Fernandez-Zamora et al., who identified significant associations between prolonged ventilation (>24 h) and a history of stroke [14]. Thanavaro et al. described predictive factors for postoperative respiratory failure, aiding in identifying patients who should be extubated as soon as possible to reduce the risk of reintubation [15]. Szylińska et al. observed that risk factors for ischemic stroke, such as atrial fibrillation, circulatory failure, and renal impairment, are also risk factors for chronic obstructive pulmonary disease [16]. Given these observations and the characteristics of the patient groups included in our study, it is reasonable to assume that the multimorbidity of these patients also increases the risk of respiratory complications, directly affecting the survival times of these patients.

In our patient group with a history of preoperative stroke undergoing isolated CABG surgery, there was a high risk of TIA incidence (OR = 4.127). Patients undergoing complex CABG surgery with valve repair exhibited a high risk of respiratory failure. Individuals undergoing valve surgery had an eight-times-higher risk of RIND occurrence (OR = 8.172). Patients undergoing aortic aneurysm repair surgery had a high risk of extensive cerebral stroke (OR = 4.387). 

The application of appropriate surgical techniques significantly influences the occurrence of complications. Letsou et al. reported a low incidence of strokes in off-pump, pump-assisted, and combined off-pump/pump-assisted coronary artery bypass grafting procedures. Perioperative mortality rates were comparable across the three techniques. The authors concluded that aortic clamping may be crucial in reducing the frequency of CABG-related strokes [17]. Svensson et al. describe pump time as the primary predictive factor for adverse events post-ascending or aortic arch operations; aortic-wall debris increases the risk of postoperative stroke [18].

Our study demonstrated an association between preoperative stroke and 30-day (OR = 1.528), 90-day (OR = 1.658), and one-year (OR = 1.706) mortality rates. The survival curve also significantly differed among patients who had experienced preoperative stroke versus those without a prior stroke history. Patients who had experienced a previous stroke exhibited significantly shorter 30-day, 90-day, and one-year survival times. Conversely, Bottle et al. observed increased mortality in patients with a history of preoperative stroke undergoing CABG [19].

Regarding the higher incidence of postoperative renal failure among patients with a history of preoperative cerebral stroke, this group had significantly higher initial creatinine concentrations. Multivariate regression analysis, which included incidence of chronic kidney disease, was used to assess the connection between preoperative cerebral stroke and postoperative complications. 

This study also revealed a statistically significant association between preoperative ischemic stroke and the occurrence of postoperative delirium. Among various neurological disorders, ischemic stroke is the most significant risk factor for delirium. Delirium poses a significant clinical challenge, especially for patients undergoing cardiac surgeries. Factors directly influencing delirium onset include systemic inflammation, pathological responses to stress, changes in synaptic activity, temperature fluctuations, hemodynamic disturbances including ischemia and reperfusion, older patient age, and the presence of other diseases that are common in cardiac patients. Therefore, obtaining an appropriate preoperative history allows the identification of patients at the highest risk of delirium, and this information can then be conveyed to the surgical and postoperative teams to aid in the targeted monitoring and management of patients affected by this condition [20,21,22].

## 5. Limitations

In addition to the limitations associated with retrospective studies, the main constraint of this work is the lack of multicenter research. Another limitation is the lack of data regarding preoperative functioning in the daily lives of patients. Routine data collection does not include information regarding the date of stroke occurrence and the outcome in terms of the NIHHS scale (National Institutes of Health Stroke Scale) and mRS (modified Rankin Scale) for patients with a history of preoperative stroke. The development of minimally invasive surgery and the qualification of patients for multidisciplinary Heart Teams have direct impacts on surgical results. The results presented in the study were also significantly influenced by the qualifications of the patients assessed by the Heart Team. Changes in pre- and postoperative fluid management and shorter times of mechanical ventilation are attributable to the cooperation of the cardiac surgeon, cardiovascular anesthesiologist, and cardiologist. 

## 6. Conclusions

Patients with a history of preoperative stroke more frequently experienced renal failure and respiratory-system complications such as pneumonia and respiratory failure. The survival time of patients with a history of preoperative stroke was shorter compared to that of the control group during the analyzed 30-day, 90-day, and one-year observation periods.

## Figures and Tables

**Table 1:** Inclusion and exclusion criteria for the study.

Inclusion Criteria	Exclusion Criteria
Patients eligible for cardiac surgery with extracorporeal circulation	urgent admission
	emergency admission
	chest injuries
	operations for atrial septal defects
	operations for ventricular septal defects
	myxoma
	pericardial sac surgeries

**Table 2:** Demographic, preoperative, and intraoperative data in patients with and without a history of preoperative stroke.

		Group I (*n* = 9283)	Group II (*n* = 546)	*p*
Demographic data				
Age [years]	mean ± SD; Me (Q1–Q3)	65.31 ± 9.48; 66.0 (60.0–72.0)	66.59 ± 8.90; 66.8 (61.0–74.0)	0.003
BMI [kg/m^2^]	mean ± SD; Me (Q1–Q3)	28.78 ± 4.44; 28.6 (25.8–31.6)	29.03 ± 4.40; 28.9 (25.9–31.9)	0.105
Sex, *n* (%)	female	2714 (29.24%)	148 (27.11%)	0.287
	male	6569 (70.76%)	398 (72.89%)	
Smoking, *n* (%)	never smoked	2477 (26.69%)	146 (26.74%)	0.995
	ex–smoker	5405 (58.24%)	317 (58.06%)	
	current smoker	1399 (15.07%)	83 (15.20%)	
Preoperative data				
Euro Score Logistic II [%], mean ± SD; Me		3.07 ± 3.75; 1.9	5.50 ± 6.42; 3.3	<0.001
EF [%], mean ± SD; Me		48.54 ± 11.32; 50.0	46.93 ± 11.45; 45.0	0.001
TIA/RIND, *n* (%)		136 (1.47%)	6 (1.10%)	0.486
EPI, *n* (%)		51 (0.55%)	4 (0.73%)	0.577
CCS 4, *n* (%)		60 (0.65%)	2 (0.37%)	0.422
NYHA III and IV, *n* (%)		2634 (28.37%)	179 (32.78%)	0.028
ICA stenosis, *n* (%)		189 (2.04%)	23 (4.21%)	<0.001
ICA occlusion, *n* (%)		14 (0.15%)	15 (2.75%)	<0.001
Carotid artery endarterectomy, *n* (%)		219 (2.36%)	87 (15.93%)	<0.001
Acute renal failure, *n* (%)		21 (0.23%)	0 (0.00%)	0.272
Chronic renal failure *n* (%)		754 (8.12%)	77 (14.10%)	<0.001
End-stage renal failure (dialysis), *n* (%)		102 (1.10%)	11 (2.01%)	0.051
Renal transplant, *n* (%)		11 (0.12%)	0 (0.00%)	0.421
Impaired insulin tolerance, *n* (%)		303 (3.26%)	12 (2.20%)	0.169
Diabetes on oral medications, *n* (%)		2678 (28.85%)	211 (38.64%)	<0.001
Diabetes on insulin, *n* (%)		972 (10.47%)	97 (17.77%)	<0.001
Arterial hypertension, *n* (%)		6641 (71.54%)	414 (75.82%)	0.032
Acute myocardial infarction, *n* (%)		26 (0.28%)	2 (0.37%)	0.713
AF paroxysmal, *n* (%)		970 (10.45%)	70 (12.82%)	0.080
AF persistent or permanent, *n* (%)		628 (6.77%)	63 (11.54%)	<0.001
Hyperlipidemia, *n* (%)		4187 (45.10%)	245 (44.87%)	0.916
COPD, *n* (%)		966 (10.41%)	55 (10.07%)	0.804
Extracardiac arteriopathy, *n* (%)		1680 (18.10%)	261 (47.80%)	<0.001
Intra- and postoperative factors				
CPB time [min], mean ± SD; Me		70.68 ± 33.88; 61.0	74.96 ± 44.07; 62.5	0.256
Aortic cross-clamping [min], mean ± SD; Me		46.52 ± 25.16; 38.0	48.52 ± 28.88; 40.0	0.487
Isolated CABG, *n* (%)		5568 (59.98%)	325 (59.52%)	0.066
CABG + valve, *n* (%)		1417 (15.26%)	104 (19.05%)	
Aortic aneurysm, *n* (%)		679 (7.31%)	36 (6.59%)	
Valve defect, *n* (%)		1619 (17.44%)	81 (14.84%)	
Time of the first intubation [hour], mean ± SD; Me		21.71 ± 70.02; 12.2	29.74 ± 86.30; 13.0	<0.001
Two intubations, *n* (%)		380 (4.09%)	43 (7.88%)	<0.001
Three or more intubations, *n* (%)		24 (0.26%)	1 (0.18%)	0.734

Legend: *n*—number of patients, SD—standard deviation, Me—median, Q1—quartile first, Q3—quartile third, BMI—body mass index, EF—ejection fraction, TIA—transient ischemic attack, RIND—reversible ischemic neurologic deficit, EPI—epilepsy, NYHA—New York Heart Association, ICA—internal carotid artery, CCS—Canadian Cardiovascular Society, AF—atrial fibrillation, COPD—chronic obstructive pulmonary disease.

**Table 3:** Assessment of laboratory data in patients with and without a history of preoperative stroke.

	Group I (*n* = 9283)			Group II (*n* = 546)			*p*
Laboratory Data	Mean	Me	SD	Mean	Me	SD	
Total WBC count (×10^9^/L)	7.67	7.35	2.82	7.71	7.42	2.21	0.424
LYMS Lymphocyte count (×10^9^/L),	2.10	1.98	1.94	2.02	1.90	0.73	0.120
Neutrophil count (×10^9^/L),	4.78	4.45	1.88	4.94	4.49	2.26	0.337
Platelets (×10^9^/L), mean ± SD	228.27	220.00	69.05	230.94	222.00	75.25	0.508
RBC (T/L)	4.51	4.54	0.52	4.45	4.50	0.55	0.049
HGB (g/dL)	8.33	8.40	1.00	8.11	8.30	1.07	<0.001
HCT (L/L)	0.40	0.40	0.04	0.39	0.40	0.05	<0.001
NLR (g/L)	2.68	2.23	1.96	2.80	2.33	2.03	0.054
PLR (g/L)	124.14	111.47	60.23	126.66	115.00	56.78	0.119
PWR (g/L)	31.32	30.03	10.47	31.03	29.90	9.77	0.979
Creatinine [mg/dL], mean ± SD	1.04	0.90	0.66	1.13	0.95	0.72	<0.001
Creatinine max [mg/dL], mean ± SD	1.38	1.07	1.00	1.56	1.24	1.02	<0.001

Legend: SD—standard deviation, Me—median, RBC—red blood cells, HGB—hemoglobin, HCT—hematocrit. WBC—white blood cell count, CRP—C-reactive protein, NLR—neutrophil−lymphocyte ratio, PLR—platelet−lymphocyte ratio, PWR—platelet−WBC ratio.

**Table 4:** Assessment of postoperative complications in patients with and without a history of preoperative stroke.

Complications after Surgery			
	Group I (*n* = 9283)	Group II (*n* = 546)	
Delirium	1454 (15.66%)	116 (21.25%)	<0.001
CVA	364 (3.92%)	30 (5.49%)	0.069
TIA	55 (0.59%)	7 (1.28%)	0.089
RIND	30 (0.32%)	3 (0.55%)	0.612
MIS	58 (0.75%)	6 (1.35%)	0.264
MAS	88 (1.13%)	7 (1.57%)	0.542
EPI	182 (1.96%)	17 (3.11%)	0.063
Myocardial infarction	311 (3.35%)	22 (4.03%)	0.394
Arrhythmias	1788 (19.26%)	115 (21.06%)	0.301
Respiratory failure	627 (6.75%)	72 (13.19%)	<0.001
Pneumonia	814 (8.77%)	86 (15.75%)	<0.001
Other pulmonary complications (pulmonary emphysema, atelectasis, bronchospasm)	572 (6.16%)	46 (8.42%)	0.043
Bleeding, tamponade	398 (4.29%)	34 (6.23%)	0.041
Sternotomy wound-healing complications	105 (1.13%)	10 (1.83%)	0.203
Healing complications of other wounds	235 (2.53%)	16 (2.93%)	0.664
Renal failure	813 (8.76%)	81 (14.84%)	<0.001
30-day mortality	386 (4.16%)	44 (8.06%)	<0.001
90-day mortality	534 (5.75%)	65 (11.90%)	<0.001
1-year mortality	734 (7.91%)	88 (16.12%)	<0.001

Legend: *n*—number of patients, CVA—cerebrovascular accident TIA—transient ischemic attack, RIND—reversible ischemic neurologic deficit, MIS-minor stroke, MAS—major stroke, EPI—epilepsy.

**Table 5:** Evaluation of the relationship between risk of postoperative complications and the occurrence of preoperative stroke.

	Model I	Model II
Delirium	1.453 (1.174–1.797) **	1.208 (0.969–1.507)
CVA	1.425 (0.972–2.089)	1.267 (0.856–1.875)
TIA	2.179 (0.988–4.808)	1.774 (0.783–4.019)
RIND	1.704 (0.518–5.601)	1.419 (0.417–4.827)
MIS	1.810 (0.777–4.218)	1.508 (0.629–3.611)
MAS	1.391 (0.640–3.021)	1.035 (0.467–2.295)
EPI	1.607 (0.970–2.662)	1.415 (0.842–2.376)
Myocardial infarction	1.211 (0.779–1.883)	1.157 (0.738–1.815)
Arrhythmias	1.118 (0.905–1.383)	1.046 (0.841–1.301)
Respiratory failure	2.097 (1.616–2.722) **	1.497 (1.141–1.964) *
Pneumonia	1.945 (1.528–2.476) **	1.482 (1.151–1.907) *
Other pulmonary complications (pulmonary emphysema, atelectasis, bronchospasm)	1.401 (1.024–1.917) *	1.342 (0.974–1.850)
Bleeding, tamponade	1.482 (1.033–2.128) *	1.285 (0.887–1.862)
Sternotomy wound-healing complications	1.631 (0.848–3.138)	1.365 (0.698–2.669)
Healing complications of other wounds	1.162 (0.695–1.943)	1.088 (0.644–1.838)
Renal failure	1.815 (1.418–2.323) **	1.391 (1.070–1.808) *
30-day mortality	2.020 (1.460–2.795) **	1.528 (1.091–2.139) *
90-day mortality	2.214 (1.684–2.910) **	1.658 (1.246–2.206) **
1-year mortality	2.238 (1.760–2.846) **	1.706 (1.327–2.194) **

Legend: CVA—cerebrovascular accident, TIA—transient ischemic attack, RIND—reversible ischemic neurologic deficit, MIS-minor stroke, MAS—major stroke, EPI—epilepsy. Notes: Model I represents univariate regression. Model II was adjusted for ESL, sex, and comorbidities. Results are presented as OR (95% CI—95% CI). Legend: * *p* < 0.05, ** *p* < 0.001

**Table 6:** Evaluation of the association between postoperative complications and preoperative stroke occurrence according to the type of surgery.

	CABG	CABG + Valve	Valve Defect	Aneurysm
Delirium	1.425 (1.071–1.895) *	1.386 (0.887–2.167)	1.646 (0.947–2.861)	1.172 (0.501–2.739)
CVA	1.186 (0.574–2.450)	1.134 (0.536–2.401)	2.052 (1.028–4.095) *	1.681 (0.570–4.954)
TIA	4.127 (1.546–11.017) *	1.000 (0.999–1.000)	2.902 (0.648–12.991)	1.000 (0.999–1.000)
RIND	1.715 (0.219–13.442)	1.000 (0.999–1.000)	8.172 (1.561–42.780) *	1.000 (0.999–1.001)
MIS	1.454 (0.342–6.183)	1.917 (0.429–8.563)	2.784 (0.625–12.410)	1.000 (0.999–1.001)
MAS	0.423 (0.053–3.084)	1.273 (0.294–5.513)	2.975 (0.664–13.330)	4.387 (0.919–20.918) *
EPI	3.609 (1.368–9.522) *	1.326 (0.518–3.394)	1.300 (0.511–3.304)	1.368 (0.313–5.981)
Myocardial infarction	0.859 (0.418–1.762)	2.047 (1.022–4.101) *	0.720 (0.172–3.005)	1.420 (0.324–6.222)
Arrhythmias	1.097 (0.828–1.454)	0.954 (0.586–1.553)	1.587 (0.958–2.630)	0.837 (0.360–1.948)
Respiratory failure	2.215 (1.522–3.223) **	2.443 (1.465–4.072) *	1.619 (0.858–3.055)	1.727 (0.647–4.611)
Pneumonia	2.178 (1.586–2.990) **	1.675 (1.013–2.769) *	1.682 (0.819–3.452)	1.361 (0.551–3.364)
Other pulmonary complications (pulmonary emphysema, atelectasis, bronchospasm)	1.249 (0.798–1.954)	1.886 (1.089–3.268) *	1.187 (0.468–3.012)	0.773 (0.180–3.316)
Bleeding, tamponade	1.575 (0.917–2.704)	1.649 (0.802–3.391)	1.859 (0.904–3.825)	0.337 (0.045–2.512)
Sternotomy wound healing complications	1.893 (0.861–4.165)	1.243 (0.288–5.361)	1.674 (0.215–13.034)	1.000 (0.999–1.000)
Healing complications of other wounds	1.394 (0.766–2.538)	0.758 (0.271–2.119)	1.000 (0.999–1.001)	1.000 (0.999–1.001)
Renal failure	1.839 (1.248–2.710) *	1.720 (1.086–2.724) *	1.631 (0.913–2.913)	2.372 (1.042–5.398) *
30-day mortality	2.290 (1.383–3.792) *	2.226 (1.261–3.928) *	1.269 (0.539–2.990)	1.804 (0.611–5.330)
90-day mortality	2.517 (1.667–3.802) **	2.106 (1.268–3.498) *	1.310 (0.617–2.780)	3.179 (1.383–7.304) *
1-year mortality	2.430 (1.712–3.451) **	2.242 (1.418–3.545) *	1.586 (0.841–2.991)	2.486 (1.091–5.665) *

Legend: CABG—coronary artery bypass grafting, CVA—cerebrovascular accident TIA—transient ischemic attack, RIND—reversible ischemic neurologic deficit, MIS-minor stroke, MAS—major stroke, EPI—epilepsy. Notes: results are presented as OR (Cl-95%—Cl + 95%). Legend: * *p* < 0.05, ** *p* < 0.001.

## Data Availability

The data can be obtained from the corresponding author upon reasonable request.
